# Late metastatic presentation is associated with improved survival and delayed wide‐spread progression after ablative stereotactic body radiotherapy for oligometastasis

**DOI:** 10.1002/cam4.4133

**Published:** 2021-08-25

**Authors:** Xuguang Chen, Hanbo Chen, Ian Poon, Darby Erler, Serena Badellino, Tithi Biswas, Roi Dagan, Matthew Foote, Alexander V. Louie, Umberto Ricardi, Arjun Sahgal, Kristin J. Redmond

**Affiliations:** ^1^ Department of Radiation Oncology and Molecular Radiation Sciences Johns Hopkins University School of Medicine Baltimore USA; ^2^ Department of Radiation Oncology Sunnybrook Odette Cancer Center University of Toronto Toronto ON Canada; ^3^ Oncology Department University of Turin Turin Italy; ^4^ University Hospitals Seidman Cancer Center Case Western Reserve University Cleveland OH USA; ^5^ Department of Radiation Oncology University of Florida Jacksonville FL USA; ^6^ Department of Radiation Oncology University of Queensland Princess Alexandra Hospital Queensland Australia

**Keywords:** late metastasis, metastasis‐directed radiotherapy, oligometastasis, SABR, SBRT, wide‐spread progression

## Abstract

**Background:**

Stereotactic body radiotherapy (SBRT) is increasingly used to treat oligometastatic disease (OMD), but the effect of metastasis timing on patient outcomes remains uncertain.

**Methods:**

An international database of patients with OMD treated with SBRT was assembled with rigorous quality assurance. Early versus late metastases were defined as those diagnosed ≤24 versus >24 months from the primary tumor. Overall survival (OS), progression‐free survival (PFS), and incidences of wide‐spread progression (WSP) were estimated using multivariable Cox proportional hazard models stratified by primary tumor types.

**Results:**

The database consists of 1033 patients with median follow‐up of 24.1 months (0.3–104.7). Late metastatic presentation (*N* = 427) was associated with improved OS compared to early metastasis (median survival 53.6 vs. 33.0 months, hazard ratio [HR] 0.59, 95% confidence interval [CI]: 0.47–0.72, *p *< 0.0001). Patients with non‐small cell lung cancer (NSCLC, *N* = 255, HR 0.49, 95% CI: 0.33–0.74, *p *= 0.0005) and colorectal cancer (*N* = 235, HR 0.50, 95% CI: 0.30–0.84, *p *= 0.008) had better OS if presenting with late metastasis. Late metastasis correlated with longer PFS (median 17.1 vs. 9.0 months, HR 0.71, 95% CI: 0.61–0.83, *p *< 0.0001) and lower 2‐year incidence of WSP (26.1% vs. 43.6%, HR 0.60, 95% CI: 0.49–0.74, *p *< 0.0001). Fewer WSP were observed in patients with NSCLC (HR 0.52, 95% CI: 0.33–0.83, *p *= 0.006) and kidney cancer (*N* = 63, HR 0.37, 95% CI: 0.14–0.97, *p *= 0.044) with late metastases. Across cancer types, greater SBRT target size was a significant predictor for worse OS.

**Conclusion:**

Late metastatic presentation is associated with improved survival and delayed progression in patients with OMD treated with SBRT.

## Graphical abstract

Despite the increasing utilization of stereotactic body radiotherapy (SBRT) for patients with oligometastasic disease (OMD), prognostic and predictive factors for this treatment modality are not well understood. In this analysis of a large, multi‐institutional database of SBRT for OMD, the timing of metastatic presentation is a significant prognostic factor, as patients with late metastasis (>24 months from cancer diagnosis) have lower risk of widespread progression and death after SBRT.

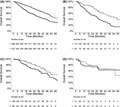

## 1 INTRODUCTION

Patients with limited metastatic disease may benefit from aggressive metastasis‐directed local therapy (MDT). Prospective clinical trials have demonstrated the efficacy of stereotactic body radiotherapy (SBRT) in patients with oligometastatic disease (OMD).^1^, ^2^, ^3^, ^4^, ^5^, ^6^ The recently updated SABR‐COMET study demonstrated significantly higher 5‐year overall survival (OS) among patients receiving SBRT than those who did not.^7^ Incorporating SBRT into the treatment paradigm of OMD may slow disease progression, delay the need to initiate or change systemic therapy, and prolong survival.^3^, ^8^, ^9^, ^10^, ^11^ However, appropriate selection criteria for patients who may benefit most from metastasis‐directed SBRT remains elusive. Relevant clinical markers, such as timing of metastatic presentation, may be useful indicators of the underlying tumor biology and inform patient selection. Previous studies offer conflicting evidence on the effect of metastasis timing on patient outcomes, with some studies suggesting a favorable effect of late metastatic presentation in patients with non‐small cell lung cancer (NSCLC), colorectal cancer, and kidney cancer,^12^, ^13^, ^14^, ^15^, ^16^, ^17^ while others showing no or the opposite effect.^18^, ^19^ In addition, the optimal threshold for stratifying patients by the timing of metastatic presentation is unclear, and past studies have used different cutoffs ranging from 1 to 36 months from the diagnosis of the primary tumor.^20^, ^21^, ^22^, ^23^ Therefore, the definition and true impact of early versus late metastasis in the setting of SBRT for OMD require further study.

This study is based on a large, international database of patients with OMD treated with SBRT at six academic institutions.^24^ OS of the entire cohort was recently reported, in which late metachronous metastatic presentation (>24 months of primary diagnosis) was associated with better OS compared to synchronous metastasis (0–6 months of primary diagnosis), while patients with early metachronous metastasis (6–24 months) had inferior OS equivalent to synchronous metastasis.^24^ The goal of the current study was to further elucidate the effect of metastasis timing on patient outcomes by additionally including progression‐free survival (PFS) and widespread progression (WSP) endpoints, and dichotomizing patients by the 24‐month threshold into “early” and “late” OMD presentations. Furthermore, since primary histology also influenced disease progression and survival, we also examined the impact of metastatic presentation on patient outcomes after stratifying by the most common primary tumor types, including NSCLC, colorectal, kidney, prostate cancer, and breast cancer, which constituted 74.3% of the entire cohort.

## 2 METHODS

### 2.1 Patients

An international, multi‐institutional database of patients with OMD treated with SBRT was constructed retrospectively using meticulous data collection and quality assurance measures, and the inclusion and exclusion criteria have been described previously.^24^ All patients had OMD, defined as 5 or fewer extra‐cranial metastases, which were treated with SBRT. Patients were excluded if they initially had more widespread disease that was downstaged to OMD after systemic therapy, presented with brain metastases or had a primary hematologic, central nervous system, or germ cell malignancy. All patients underwent complete baseline staging, including CT and/or MRI of the brain, CT of the chest/abdomen/pelvis and/or PET scan, within 4 months of the first SBRT treatment.

### 2.2 Treatment and follow‐up

Stereotactic body radiotherapy treatments were delivered in fewer than 10 fractions using stereotactic technique. Patients receiving palliative fractionation schemes such as 4 Gy ×5 fractions, 3 Gy ×10 fractions, or 8 Gy ×1 or 2 fractions were excluded. Detailed dose and fractionation schemes stratified by treatment sites have been previously reported.^24^ The mean total dose was 41.8 Gy and the mean number of fractions was 4.6 (median 5). Site‐specific simulation, immobilization, and image guidance were determined by each participating institution and described previously.^25^ Post‐SBRT follow‐up was performed in a treatment site and institution‐specific manner, and consisted of at least a CT chest, abdomen, and pelvis every 2–4 months for the first year, 3–6 months in year 2–4, and 6–12 months in year 5 and thereafter.^26^

### 2.3 Clinical variables and outcomes

Based on our previous study, early versus late metastatic presentations were defined as the first metastasis diagnosed ≤24 months versus >24 months from the diagnosis of the primary tumor. Other clinical variables include the total number of metastases, whether or not all metastases were confined to the bone (bone‐only metastasis), the total size of the SBRT planning target volume (PTV, in cm^3^) during the first course of SBRT, and the total biologically effective dose at *α*/*β* of 10 (BED_10_) during the first course of SBRT. Genomic and biologic variables were also included when applicable, including EGFR and ALK mutation status in NSCLC, KRAS mutation status in colorectal cancer, Gleason score and pre‐SBRT PSA level for prostate cancer, and tumor grade and receptor subtype for breast cancer. The main outcomes of this study were OS, PFS, and incidence of WSP. Progression was defined as radiographic evidence of either local progression, new metastasis, death, or last follow‐up for patients who were alive. WSP was defined as the development of ≥6 new sites of extracranial metastases or wide dissemination that precluded further local ablative therapy, such as malignant effusion.

### 2.4 Statistical analysis

Overall survival and PFS rates were estimated by the Kaplan–Meier method. The start of follow‐up was defined as the start date of the first course of SBRT for all patients to allow for the same baseline and avoid immortal time bias between time of diagnosis and the first course of SBRT. Univariable and multivariable Cox regressions for OS and PFS were performed for the entire patient cohort, as well as in each of the five most common primary tumor types to estimate the effect of prognostic factors listed in the previous section. WSP rates were estimated by the cumulative incidence function using death as a competing risk. Univariable and multivariable Fine and Gray competing risks regressions were used to estimate the effect of the prognostic factors on WSP for the entire cohort as well as each of the five primary tumor types.^27^ All tests were two‐tailed, and results were considered significant if the *p* value was <0.05. Statistical analyses were performed using R (x64, v4.0.2).^28^

## 3 RESULTS

The entire cohort consisted of 1033 patients with OMD treated with SBRT, with a median follow‐up time of 24.1 months (range: 0.3–104.7). The five most common primary tumor types were NSCLC (*N* = 255, 24.7%), colorectal cancer (*N* = 235, 22.7%), prostate cancer (*N* = 132, 12.8%), breast cancer (*N* = 83, 8.0%), and kidney cancer (*N* = 63, 6.1%). Table 1 summarizes the baseline patient and treatment characteristics. The median age at the start of SBRT was 66.8 years (standard deviation [SD]: 12.8), and 601 patients (58.2%) were male. The majority of patients in the entire cohort (58.7%), as well as those with NSCLC (67.8%) and colorectal cancer (63.4%), had early metastatic presentation (≤24 months), while the majority of patients with prostate cancer and breast cancer had late metastasis (66.7% and 55.4%, respectively). The majority of patients (57.1%) had only one metastasis at the time of presentation, and 91.4% had three or fewer metastases. Ninety‐five percent of patients had all known sites of OMD treated upfront. There was a wide range of total PTV sizes during the first course of SBRT (Mean ± SD: 66.7 ± 75.6 cm^3^), and patients with kidney cancers appeared to have the greatest target volume (Mean ± SD: 110.5 ± 116.1 cm^3^). The mean total SBRT prescription BED_10_ during the first course of SBRT was 114.7 Gy_10_ (SD: 72.1 Gy_10_).

**TABLE 1:** Baseline patient and treatment characteristics

	Total	NSCLC	Colorectal	Kidney	Prostate	Breast
*N* (%)	1033 (100.0)	255 (24.7)	235 (22.7)	63 (6.1)	132 (12.8)	83 (8.0)
Age (Mean ± SD)	66.8 ± 12.8	70.2 ± 10.6	70.6 ± 12.2	66.8 ± 10.4	70.2 ± 7.6	55.0 ± 11.0
Male (%)	601 (58.2)	137 (53.7)	140 (59.6)	39 (61.9)	132 (100.0)	1 (1.2)
Metastasis timing (%)						
Early (≤24 months)	606 (58.7)	173 (67.8)	149 (63.4)	31 (49.2)	44 (33.3)	37 (44.6)
Late (>24 months)	427 (41.3)	82 (32.2)	86 (36.6)	32 (50.8)	88 (66.7)	46 (55.4)
Total no. of metastases						
1	590 (57.1)	193 (75.7)	95 (40.4)	30 (47.6)	80 (60.6)	49 (59.0)
2	248 (24.0)	43 (16.9)	62 (26.4)	22 (34.9)	26 (19.7)	23 (27.7)
3	106 (10.3)	13 (5.1)	40 (17.0)	6 (9.5)	13 (9.8)	7 (8.4)
4	57 (5.5)	4 (1.6)	23 (9.8)	4 (6.3)	8 (6.1)	4 (4.8)
5	32 (3.1)	2 (0.8)	15 (6.4)	1 (1.6)	5 (3.8)	0 (0.0)
Total PTV volume in cm^3^ (Mean ± SD)	66.7 ± 75.6	57.0 ± 70.0	61.3 ± 71.1	110.5 ± 116.1	61.8 ± 61.7	69.6 ± 73.0
Total BED_10_ in Gy (Mean ± SD)	114.7 ± 72.1	108.5 ± 51.5	135.8 ± 74.0	99.4 ± 69.0	106.0 ± 82.8	83.7 ± 47.8
Pre‐SBRT systemic therapy (%)	368 (35.6)	46 (18.0)	96 (40.9)	11 (17.5)	82 (62.1)	56 (67.5)

Abbreviations: BED_10_, biologically effective dose at *α*/*β* ratio of 10 Gy; NSCLC, non‐small cell lung cancer; PTV, planning target volume; SD, standard deviation.

**FIGURE 1:**
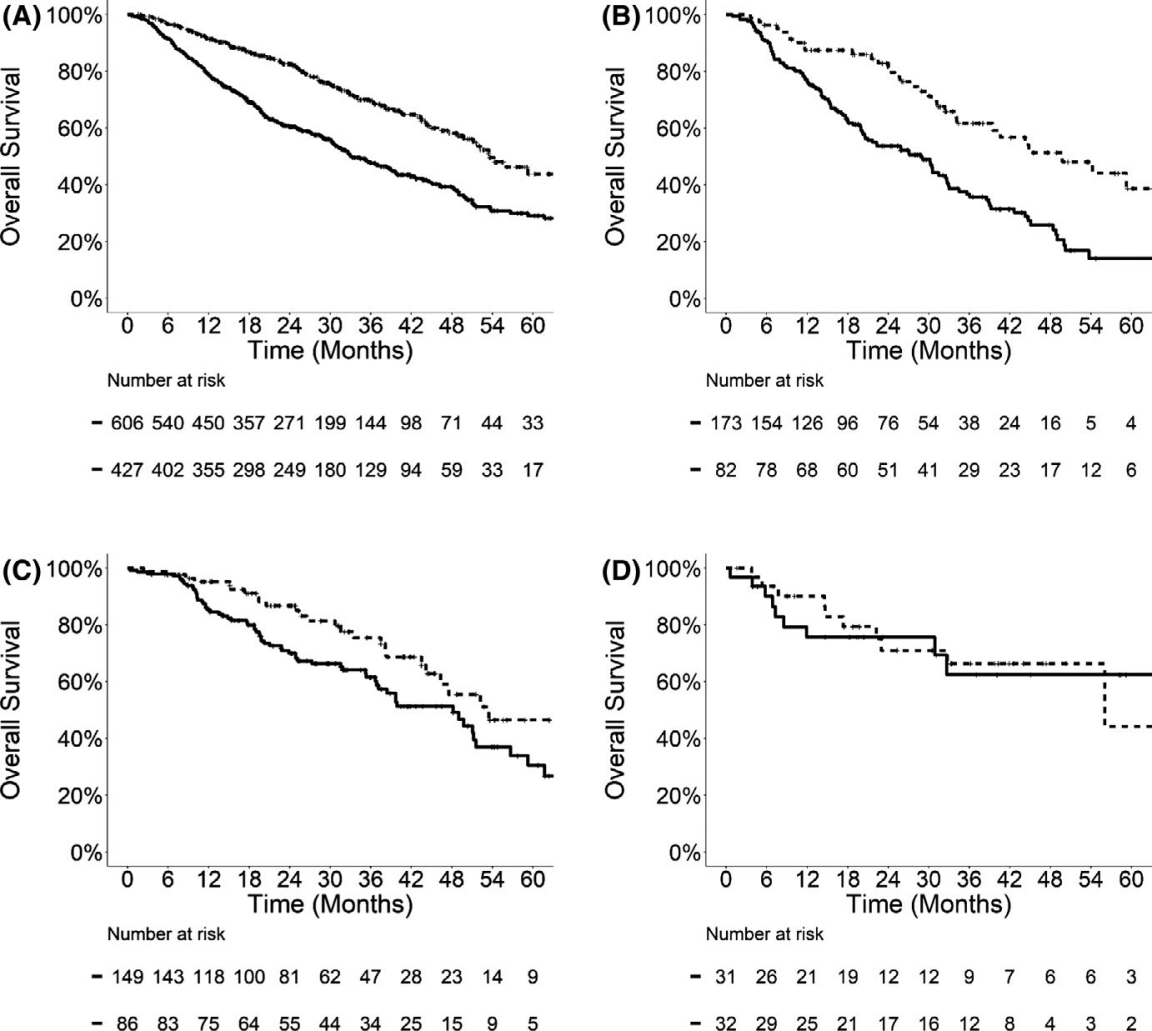
Kaplan–Meier survival curves for overall survival in the entire cohort (A), and patients with NSCLC (B), colorectal cancer (C), and kidney cancer (D). Solid lines, early metastasis (diagnosed ≤24 months of the primary tumor); dashed lines, late metastasis (diagnosed >24 months after the primary tumor)

Univariable regression analysis (Figure 1) revealed that late metastatic presentation was associated with significantly better OS in the entire cohort (HR 0.51, 95% CI: 0.42–0.63, *p *< 0.0001), as well as in patients with NSCLC (HR 0.46, 95% CI: 0.31–0.68, *p *= 0.0001) and colorectal cancer (HR 0.59, 95% CI: 0.38–0.94, *p *= 0.025). In the overall cohort, the median OS for patients with late metastatic presentation was 53.6 months (95% CI: 49.8–70.0), significantly longer than those with early metastasis (33.0 months, 95% CI: 30.6–39.2). When stratified by the primary tumor type, median OS was also significantly longer for late metastasis among patients with NSCLC (49.8 months, 95% CI: 39.4‐not reached vs. 28.8 months, 95% CI: 20.5–32.7) and colorectal cancer (53.5 months, 95% CI: 46.3‐not reached vs. 48.2 months, 95% CI: 37.2–56.7). The effect of late metastatic presentation on OS remained significant in multivariable analyses after adjusting for the total number of metastases, bone‐only (vs. soft tissue) metastasis, total PTV size, total SBRT BED_10_, and tumor mutational status. Table 2 demonstrates the results of multivariable analyses for the entire cohort (HR 0.59 for late vs. early metastasis, 95% CI: 0.47–0.72, *p *< 0.0001), NSCLC (HR 0.49, 95% CI: 0.33–0.74, *p *= 0.0005), and colorectal cancer (HR 0.50, 95% CI: 0.30–0.84, *p *= 0.008). There was no statistically significant difference in OS between early versus late metastasis in patients with renal cell carcinoma (HR 1.24, 95% CI: 0.45–3.44, *p *= 0.68).

**TABLE 2:** Multivariable analysis of factors associated with overall survival

	Entire cohort^a^		NSCLC		Colorectal		Kidney	
	HR (95% CI)	*p* value	HR (95% CI)	*p* value	HR (95% CI)	*p* value	HR (95% CI)	*p* value
Late metastatic presentation	0.59 (0.47–0.72)	<0.0001^##^	0.49 (0.33–0.74)	0.0005^##^	0.50 (0.30–0.84)	0.008^#^	1.24 (0.45–3.44)	0.68
Total # metastases	1.06 (0.95–1.18)	0.33	0.92 (0.68–1.24)	0.58	1.16 (0.95–1.41)	0.14	1.42 (0.84–2.43)	0.19
Bone‐only metastasis	0.93 (0.69–1.25)	0.63	1.63 (0.97–2.73)	0.063	5.06 (1.47–17.4)	0.01*	0.54 (0.13–2.27)	0.4
Total PTV volume	1.003 (1.002–1.004)	<0.0001^##^	1.002 (1.000–1.004)	0.019*	1.003 (1.001–1.005)	0.005^#^	1.004 (1.000–1.008)	0.029*
Total BED_10_	0.999 (0.998–1.001)	0.44	1.000 (0.995–1.005)	0.89	1.000 (0.997–1.003)	0.95	0.994 (0.986–1.002)	0.14
EGFR mutation	NA	NA	0.82 (0.28–2.39)	0.72	NA	NA	NA	NA
ALK mutation	NA	NA	0.24 (0.03–1.86)	0.17	NA	NA	NA	NA
KRAS mutation	NA	NA	NA	NA	0.60 (0.29–1.22)	0.16	NA	NA

Abbreviations: BED_10_, biologically effective dose at *α*/*β* ratio of 10 Gy; CI, confidence interval; HR, hazard ratio.

^a^ Multivariable model for the entire cohort also adjusted for primary tumor type.

* *p* < 0.05; #*p *< 0.01; ##*p *< 0.001.

**FIGURE 2:**
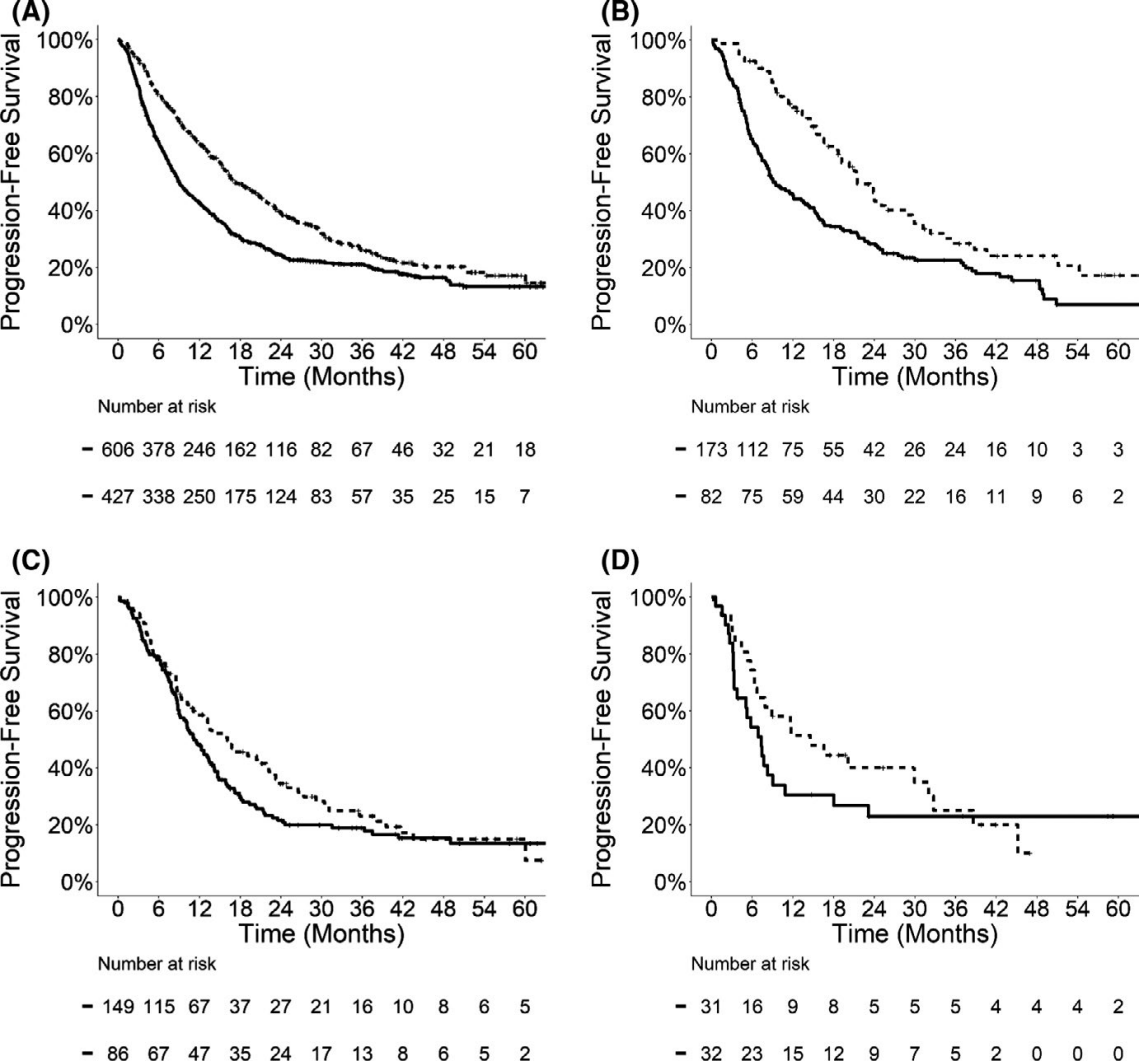
Kaplan–Meier survival curves for progression‐free survival in the entire cohort (A), and patients with NSCLC (B), colorectal cancer (C), and kidney cancer (D). Solid lines, early metastasis (diagnosed ≤24 months of the primary tumor); dashed lines, late metastasis (diagnosed >24 months after the primary tumor)

**FIGURE 3:**
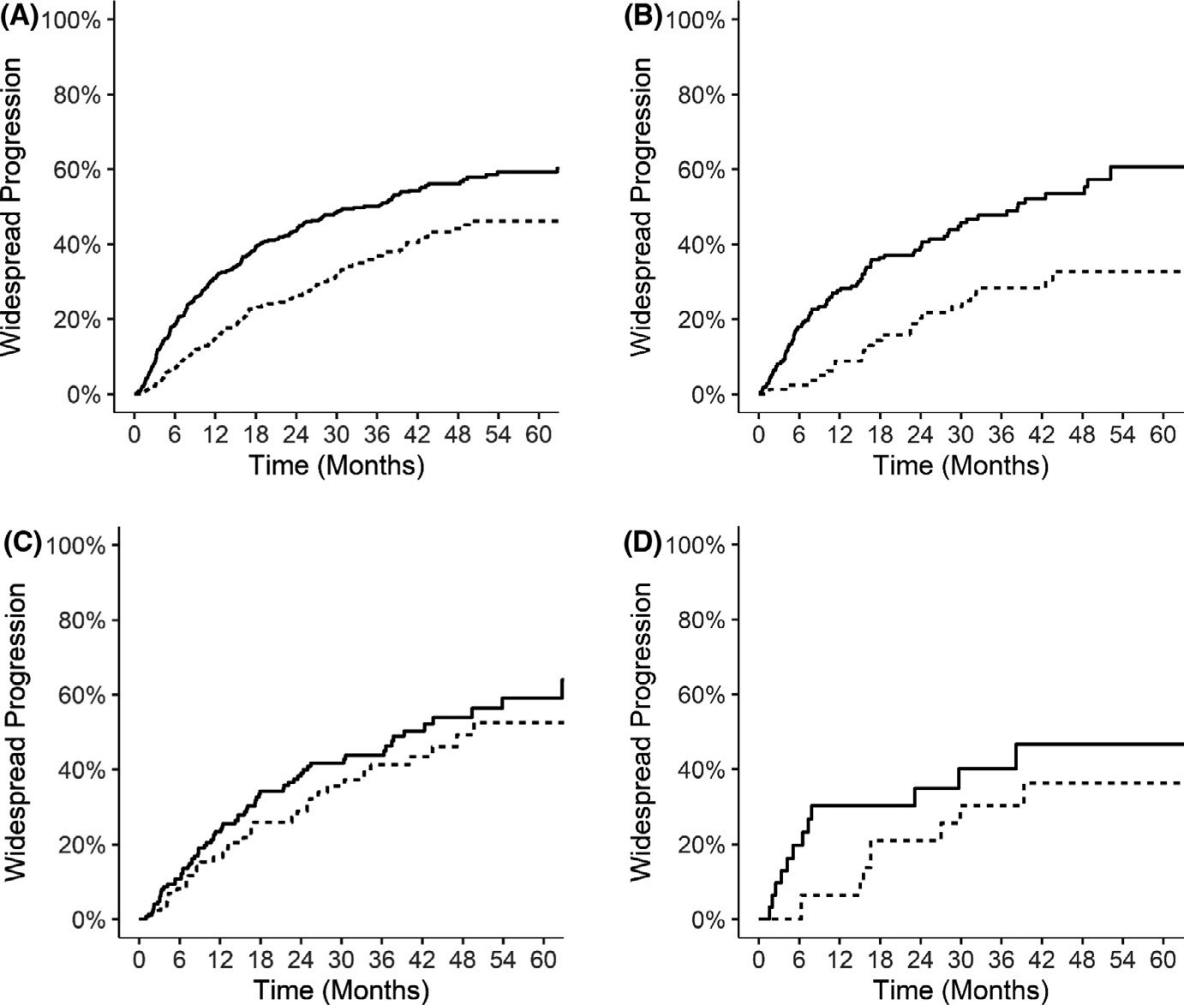
Kaplan–Meier failure curves for wide‐spread progression in the entire cohort (A), and patients with NSCLC (B), colorectal cancer (C), and kidney cancer (D). Solid lines, early metastasis (diagnosed ≤24 months of the primary tumor); dashed lines, late metastasis (diagnosed >24 months after the primary tumor)

To understand whether the superior OS in patients with late metastatic presentation was due to delayed disease progression, we examined PFS (Figure 2; Table 3) and incidences of WSP (Figure 3; Table 4) in relation to metastasis timing. Patients with late metastatic presentation demonstrated prolonged median PFS compared to those with early metastasis in the entire cohort (17.1 vs. 9.0 months, *p *< 0.0001), NSCLC (21.5 vs. 9.5 months, *p *= 0.0002), colorectal cancer (16.1 vs. 11.2 months, *p *= 0.14), and kidney cancer (14.6 vs. 7.3 months, *p *= 0.38). On multivariable analyses, late metastatic presentation was associated with significantly longer PFS in the entire cohort (HR 0.71, 95% CI: 0.61–0.83, *p *< 0.0001) and patients with NSCLC (HR 0.60, 95% CI: 0.43–0.83, *p *= 0.002), and a trend toward longer PFS in patients with colorectal cancer (HR 0.77, 95% CI: 0.56–1.07, *p *= 0.12).

**TABLE 3:** Multivariable analysis of factors associated with progression‐free survival

	Entire cohort^a^		NSCLC		Colorectal		Kidney	
	HR (95% CI)	*p* value	HR (95% CI)	*p* value	HR (95% CI)	*p* value	HR (95% CI)	*p* value
Late metastatic presentation	0.71 (0.61–0.83)	<0.0001^##^	0.60 (0.43–0.83)	0.002^#^	0.77 (0.56–1.07)	0.12	0.74 (0.40–1.38)	0.35
Total # metastases	1.11 (1.02–1.20)	0.011*	1.07 (0.84–1.37)	0.59	1.05 (0.91–1.20)	0.52	1.27 (0.89–1.81)	0.2
Bone‐only metastasis	1.22 (0.98–1.52)	0.082	1.21 (0.77–1.89)	0.42	1.08 (0.33–3.48)	0.90	1.90 (0.78–4.59)	0.16
Total PTV volume	1.003 (1.002–1.004)	<0.0001^##^	1.006 (1.004–1.008)	<0.0001^##^	1.002 (1.000–1.004)	0.022*	1.001 (0.998–1.004)	0.52
Total BED_10_	1.000 (0.998–1.000)	0.46	0.999 (0.995–1.003)	0.51	1.002 (1.000–1.005)	0.029*	1.000 (0.996–1.005)	0.86
EGFR mutation	NA	NA	0.97 (0.38–2.51)	0.95	NA	NA	NA	NA
ALK mutation	NA	NA	1.20 (0.35–4.15)	0.77	NA	NA	NA	NA
KRAS mutation	NA	NA	NA	NA	1.10 (0.70–1.74)	0.68	NA	NA

Abbreviations: BED_10_, biologically effective dose at *α*/*β* ratio of 10 Gy; CI, confidence interval; HR, hazard ratio.

^a^ Multivariable model for the entire cohort also adjusted for primary tumor type.

* *p* < 0.05; #*p *< 0.01; ##*p *< 0.001.

**TABLE 4:** Multivariable analysis of factors associated with wide‐spread progression

	Entire cohort^a^		NSCLC		Colorectal		Kidney	
	HR (95% CI)	*p* value	HR (95% CI)	*p* value	HR (95% CI)	*p* value	HR (95% CI)	*p* value
Late metastatic presentation	0.60 (0.49–0.74)	<0.0001^##^	0.52 (0.33–0.83)	0.006^#^	0.71 (0.46–1.10)	0.12	0.37 (0.14–0.97)	0.044*
Total # metastases	1.18 (1.07–1.30)	0.001^#^	1.09 (0.80–1.48)	0.57	1.26 (1.08–1.48)	0.004^#^	2.22 (1.33–3.70)	0.002^#^
Bone‐only metastasis	1.40 (1.04–1.90)	0.029	2.38 (1.36–4.17)	0.002^#^	1.52 (0.22–10.6)	0.67	14.1 (5.51–36.1)	<0.0001^##^
Total PTV volume	1.002 (1.001–1.004)	<0.0001^##^	1.003 (1.000–1.006)	0.026*	1.003 (1.000–1.010)	0.027*	0.997 (0.993–1.001)	0.13
Total BED_10_	1.000 (0.999–1.002)	0.50	1.000 (0.995–1.006)	0.87	1.003 (1.001–1.005)	0.008^#^	1.002 (0.998–1.007)	0.31
EGFR mutation	NA	NA	0.95 (0.23–3.99)	0.95	NA	NA	NA	NA
ALK mutation	NA	NA	1.49 (0.62–3.57)	0.37	NA	NA	NA	NA
KRAS mutation	NA	NA	NA	NA	1.18 (0.77–1.83)	0.44	NA	NA

Abbreviations: BED_10_, biologically effective dose at *α*/*β* ratio of 10 Gy; CI, confidence interval; HR, hazard ratio.

^a^ Multivariable model for the entire cohort also adjusted for primary tumor type.

* *p* < 0.05; #*p *< 0.01; ##*p *< 0.001.

We also observed lower risk of developing WSP in patients with late metastatic presentation. At 2 years after SBRT, the incidence of WSP was 26.1% (95% CI: 21.6%–30.5%) versus 43.6% (95% CI: 39.5%–47.7%) for patients with late versus early metastasis in the entire cohort (*p *< 0.0001). When stratified by primary tumor type, the 2‐year incidence of WSP was also lower for late metastasis in patients with NSCLC (20.3%, 95% CI: 11.0%–29.7%), colorectal cancer (28.9%, 95% CI: 18.7%–39.1%), and kidney cancer (21.0%, 95% CI: 5.7%–36.3%) than those with early metastasis in the NSCLC (39.3%, 95% CI: 31.7%–46.8%, *p *= 0.0004), colorectal cancer (38.3%, 95% CI: 30.0%–46.5%, *p *= 0.22), and kidney cancer (34.9%, 95% CI: 16.6%–53.2%, *p *= 0.17) subgroups. On multivariable analyses, late metastatic presentation was a significant predictor for decreased incidence of WSP in the entire cohort (HR 0.60, 95% CI: 0.49–0.74, *p *< 0.0001), as well as patients with NSCLC (HR 0.52, 95% CI: 0.33–0.83, *p *= 0.006) and kidney cancer (HR 0.37, 95% CI: 0.14–0.97, *p *= 0.044). The HR for late metastasis was 0.71 (95% CI: 0.46–1.10, *p *= 0.12) among patients with colorectal cancer.

In patients with prostate cancer or breast cancer, the timing of metastatic presentation was not a significant predictor for OS, PFS, or WSP (Tables S1 and S2; Figures S1 and S2). On the other hand, total PTV size during the first course of SBRT was significantly associated with worse OS in the entire cohort (HR 1.003 per cm^3^ increase, 95% CI: 1.002–1.004, *p *< 0.0001) and all the subgroups (HR 1.002–1.017), indicating a relationship between initial metastatic volume and OS. The effect of total PTV size on OS was most pronounced in patients with prostate cancer (HR 1.017 per cm^3^ increase, 95% CI: 1.004–1.031). This effect was also observed in relation to PFS and WSP in the entire cohort, as well as patients with NSCLC, colorectal cancer, and prostate cancer (Tables 3 and 4; Table S1). In contrast, the total absolute number of metastases was not associated with survival after SBRT.

## 4 DISCUSSION

In this study, we defined a single clear threshold for stratifying patients with OMD by the timing of metastatic presentation. Using the definition of early (≤24 months of primary tumor diagnosis) versus late metastasis (>24 months), metastatic presentation was a significant predictor for disease progression and survival after SBRT for OMD in a large, diverse, and well‐characterized patient cohort. These data can improve risk stratification and prognostication for patients with OMD being considered for SBRT, and ultimately advance our understanding of what subset of patients may be potentially curable despite the development of metastases.

Most studies to date have reported a positive association between late metastatic presentation and better patient outcome. For example, in a retrospective cohort of 309 patients with OMD treated with SBRT, metastasis diagnosed within 1 month of the primary tumor was an independent predictor of worse OS compared to metastasis diagnosed after 1 month.^20^ Similar association between metastasis timing and patient survival and disease control after SBRT has been observed for NSCLC, colorectal cancer, and kidney cancer, as well as oligometastasis to the lung and liver.^12^, ^13^, ^14^, ^16^, ^17^, ^29^ Several studies, however, have noted no or the opposite effect of metastasis timing on survival and disease progression in patients with NSCLC or pulmonary oligometastasis.^18^, ^19^ This discrepancy may be partly due to the various thresholds used to define synchronous versus metachronous metastases, ranging from 1 to 6 months of the diagnosis of the primary tumor.^12^, ^19^, ^20^, ^21^ Other studies using longer thresholds (24–36 months) for disease‐free interval (DFI) have demonstrated positive association between longer DFI and improved patient outcomes in patients with pulmonary oligometastasis after SBRT or metastatectomy.^22^, ^23^, ^30^ These data suggest a need to better define the threshold for stratifying patients with OMD based on metastasis timing.

In the current study, we chose a threshold of 24 months to define early versus late metastatic presentations based on multiple rationales. First, as previous report of our entire cohort demonstrated, patients with synchronous metastasis (diagnosed within 6 months of the primary tumor) had equivalent OS to those with early metachronous metastasis (6–24 months after primary diagnosis), and both groups had inferior survival compared to patients with late metachronous metastases (>24 months after primary diagnosis).^24^ Second, when stratified by primary tumor type, we observed similar OS in patients with synchronous (0–6 months) and early metachronous metastases (6–24 months after primary diagnosis) for NSCLC, colorectal, kidney, prostate cancer, and breast cancer (Figure S3). Third, the difference between late and early metastases in the current study is similar to the difference between metachronous and synchronous metastases in previous reports. For example, the HR for late versus early metastasis was 0.50 in our cohort of patients with NSCLC, while the HR of OS for metachronous timing (vs. synchronous timing) was 0.51 in one of the largest series of patients with oligometastatic NSCLC.^12^ Similarly, the HR for WSP was 0.37 for late versus early metastasis in our cohort of patients with kidney cancer, while metachronous timing was associated with prolonged freedom from systemic therapy after SBRT (HR of 0.37) in patients with renal cell carcinoma.^8^ Finally, the effect of late versus early metastasis timing appeared consistent across multiple primary tumor types, including NSCLC, colorectal cancer, and kidney cancer. Taken together, our data suggest that 24 months is a superior threshold for predicting patient outcomes based on the timing of metastatic presentation.

We also comprehensively investigated the relationship between metastasis timing and WSP, a novel outcome that may have important clinical utility. In our series, late metastasis was associated with reduced incidences of WSP in the entire cohort (*p *< 0.0001), and in patients with NSCLC (*p *= 0.006), kidney cancer (*p *= 0.044), and potentially colorectal cancer (*p* = 0.12), suggesting that these patients have the lowest risk of converting to systemic disease, thus may benefit most from MDT. However, SBRT for OMD resulted in excellent patient outcomes, even among those who had early metastatic presentation. In patients presenting with early metastatic disease, the median OS was nearly 3 years (33 months), and less than half (43.6%) developed WSP at 2 years, indicating that the majority of patients in this subgroup may still benefit from local therapy, such as SBRT. Given the excellent tumor control and low rate of toxicity, SBRT remains an appropriate therapeutic option in patients presenting with early or synchronous metastasis. Moreover, these data support the use of repeated SBRT for patients who develop oligoprogression after initial SBRT. Collectively, our findings are consistent with the ESTRO‐ASTRO consensus statement that metastasis timing may define different types of OMD but a DFI is not necessary for patients to be considered for SBRT.^31^

Initial PTV size, rather than the total number of metastases, was associated with poor survival in our study. Of note, we used PTV size, instead of gross tumor volume (GTV) or clinical target volume (CTV), in the multivariable analyses, because PTV data were more complete (only 2.3% data points missing) than GTV/CTV (10.9% missing). Given PTV is typically a limited uniform expansion of GTV/CTV, we are confident that PTV size is a reasonable surrogate for tumor volume. Similar effect of PTV size on local control and survival has been observed in previous studies. For example, in a retrospective series of 317 patients with pulmonary oligometastasis, PTV size was associated with local control after SBRT.^32^ PTV size was also associated with poorer local control and/or OS in patients with oligometastatic ovarian and colorectal cancer.^33^, ^34^, ^35^ These observations may be explained by the presence of a large number of active tumor cells, represented by greater PTV size, increases the likelihood of metastatic dissemination, and lowers the effectiveness of tumor‐directed therapy. Whether SBRT dose escalation or combining SBRT with more active systemic agents, such as immunotherapy, could overcome treatment resistance remains to be determined.

We did not observe a clear association between metastasis timing and patient outcomes with prostate cancer or breast cancer, possibly due to a multitude of factors. First, patients with oligometastatic prostate cancer and breast cancer had much better outcome in our dataset, with 2‐year OS rate of 96.0% and 89.7%, respectively. As a result, there were fewer events observed in these subgroups, limiting the statistical power to detect a difference. Second, patients with prostate cancer and breast cancer may have more active systemic therapy options, since both are largely hormone‐driven malignancies. Lastly, given the superior outcome and more indolent natural history, a threshold much longer than 24 months may be needed to classify early versus late metastatic presentation for patients with prostate cancer and breast cancer.

Given the variable natural history of different cancer types in our study, consideration of SBRT for OMD needs to be individualized based on each patient's history, disease burden, and personal values. For patients with lower risk of systemic spread, for example, those with prostate cancer, kidney cancer, or late‐presenting oligometastatic NSCLC, initial SBRT may be favored instead of systemic therapy, especially the types of systemic therapies that have higher risks of toxicities. In contrast, patients at higher risk of systemic progression, such as those with early presenting oligometastatic NSCLC, colorectal cancer, or breast cancer, may benefit from early initiation of systemic therapy in conjunction with SBRT. Future prospective randomized trials may provide further guidance on patient selection and SBRT timing.

In conclusion, in this large international multi‐institutional cohort of patients with OMD, late metastatic presentation (>24 months from the diagnosis of the primary tumor) is associated with better overall survival and decreased incidence of wide‐spread progression. This effect was evident in multiple cancer types, including NSCLC, colon cancer, and kidney cancer, suggesting metastasis timing is a true marker for disease aggressiveness. Absolute tumor burden, as measured by SBRT target size, is a negative predictor of overall survival in patients with NSCLC, colorectal, kidney, prostate cancer, and breast cancer. Patients with both early and late oligometastases should be considered for metastasis‐directed SBRT given the favorable outcomes in our cohort. Future prospective trials of SBRT for OMD should consider stratifying patients based on primary tumor type and timing of metastatic presentation.

## CONFLICT OF INTEREST

X.C., H.C., and U.R. have no conflict of interest to declare. I.P., D.E., S.B., T.B., and R.D. have received travel support from Elekta AB. M.F. has received honoraria, research grant, and travel expenses from Elekta AB. A.V.L. has received honoraria from Varian medical systems, AstraZeneca, and RefleXion. A.S. has done educational seminars with Medtronic, Elekta AB, Accuray Inc., and Varian medical systems, has received research grants and travel expenses from Elekta AB, and is part of the Elekta MR‐Linac research consortium. K.J.R. has received research support, travel expenses, and honorarium for educational seminars from Accuray; participates in a data safety monitoring committee for BioMimetix; and received travel expenses from Brainlab and Elekta AB; and received honorarium for speaking engagement from NCCN.

## Supporting information

Supplementary Material

## Data Availability

The data that support the findings of this study are available upon request from the corresponding author. The data are not publicly available due to privacy or ethical restrictions.

## References

[1] Gomez DR, Blumenschein GR, Lee JJ, et al. Local consolidative therapy versus maintenance therapy or observation for patients with oligometastatic non‐small‐cell lung cancer without progression after first‐line systemic therapy: a multicentre, randomised, controlled, phase 2 study. Lancet Oncol. 2016;17(12):1672‐1682. 10.1016/s1470-2045(16)30532-0 27789196 PMC5143183

[2] Gomez DR, Tang C, Zhang J, et al. Local consolidative therapy vs. maintenance therapy or observation for patients with oligometastatic non‐small‐cell lung cancer: long‐term results of a multi‐institutional, phase II, randomized study. J Clin Oncol. 2019;37(18):1558‐1565. 10.1200/jco.19.00201 31067138 PMC6599408

[3] Ost P, Reynders D, Decaestecker K, et al. Surveillance or metastasis‐directed therapy for oligometastatic prostate cancer recurrence: a prospective, randomized, multicenter phase II trial. J Clin Oncol. 2018;36(5):446‐453. 10.1200/jco.2017.75.4853 29240541

[4] Phillips R, Shi WY, Deek M, et al. Outcomes of observation vs stereotactic ablative radiation for oligometastatic prostate cancer: the ORIOLE phase 2 randomized clinical trial. JAMA Oncol. 2020;6(5):650‐659. 10.1001/jamaoncol.2020.0147 32215577 PMC7225913

[5] Chen H, Louie AV, Higginson DS, Palma DA, Colaco R, Sahgal A. Stereotactic radiosurgery and stereotactic body radiotherapy in the management of oligometastatic disease. Clin Oncol (R Coll Radiol). 2020;32(11):713‐727. 10.1016/j.clon.2020.06.018 32718762

[6] Palma DA, Louie AV, Rodrigues GB. New strategies in stereotactic radiotherapy for oligometastases. Clin Cancer Res. 2015;21(23):5198‐5204. 10.1158/1078-0432.ccr-15-0822 26626571

[7] Palma DA, Olson R, Harrow S, et al. Stereotactic ablative radiotherapy for the comprehensive treatment of oligometastatic cancers: long‐term results of the SABR‐COMET phase II randomized trial. J Clin Oncol. 2020;38(25):2830‐2838. 10.1200/JCO.20.00818 32484754 PMC7460150

[8] Zhang Y, Schoenhals J, Christie A, et al. Stereotactic ablative radiation therapy (SAbR) used to defer systemic therapy in oligometastatic renal cell cancer. Int J Radiat Oncol Biol Phys. 2019;105(2):367‐375. 10.1016/j.ijrobp.2019.07.023 31377159 PMC7647381

[9] Merino Lara T, Helou J, Poon I, et al. Multisite stereotactic body radiotherapy for metastatic non‐small‐cell lung cancer: delaying the need to start or change systemic therapy? Lung Cancer. 2018;124:219‐226. 10.1016/j.lungcan.2018.08.005 30268464

[10] Deek MP, Taparra K, Phillips R, et al. Metastasis‐directed therapy prolongs efficacy of systemic therapy and improves clinical outcomes in oligoprogressive castration‐resistant prostate cancer. Eur Urol Oncol. 2020;108(3):e893‐e894. 10.1016/j.euo.2020.05.004 PMC7788526 32536574

[11] Friedes C, Mai N, Fu W, et al. Isolated progression of metastatic lung cancer: clinical outcomes associated with definitive radiotherapy. Cancer. 2020;126(20):4572‐4583. 10.1002/cncr.33109 32729962

[12] Ashworth AB, Senan S, Palma DA, et al. An individual patient data metaanalysis of outcomes and prognostic factors after treatment of oligometastatic non‐small‐cell lung cancer. Clin Lung Cancer. 2014;15(5):346‐355. 10.1016/j.cllc.2014.04.003 24894943

[13] Fode MM, Høyer M. Survival and prognostic factors in 321 patients treated with stereotactic body radiotherapy for oligo‐metastases. Radiother Oncol. 2015;114(2):155‐160. 10.1016/j.radonc.2014.12.003 25583567

[14] Wong AC, Watson SP, Pitroda SP, et al. Clinical and molecular markers of long‐term survival after oligometastasis‐directed stereotactic body radiotherapy (SBRT). Cancer. 2016;122(14):2242‐2250. 10.1002/cncr.30058 27206146

[15] Friedes C, Mai N, Hazell S, et al. Consolidative radiotherapy in oligometastatic lung cancer: patient selection with a prediction nomogram. Clin Lung Cancer. 2020;21(6):e622‐e632. 10.1016/j.cllc.2020.05.013 32624411

[16] Agolli L, Bracci S, Nicosia L, Valeriani M, De Sanctis V, Osti MF. Lung metastases treated with stereotactic ablative radiation therapy in oligometastatic colorectal cancer patients: outcomes and prognostic factors after long‐term follow‐up. Clin Colorectal Cancer. 2017;16(1):58‐64. 10.1016/j.clcc.2016.07.004. 27522627

[17] Franzese C, Franceschini D, Di Brina L, et al. Role of stereotactic body radiation therapy for the management of oligometastatic renal cell carcinoma. J Urol. 2019;201(1):70‐75. 10.1016/j.juro.2018.08.049 30577391

[18] Fleckenstein J, Petroff A, Schäfers HJ, Wehler T, Schöpe J, Rübe C. Long‐term outcomes in radically treated synchronous vs. metachronous oligometastatic non‐small‐cell lung cancer. BMC Cancer. 2016;16:348. 10.1186/s12885-016-2379-x PMC4890277 27255302

[19] Sharma A, Duijm M, Oomen‐de Hoop E, et al. Survival and prognostic factors of pulmonary oligometastases treated with stereotactic body radiotherapy. Acta Oncol. 2019;58(1):74‐80. 10.1080/0284186x.2018.1521986 30280633

[20] de Vin T, Engels B, Gevaert T, Storme G, De Ridder M. Stereotactic radiotherapy for oligometastatic cancer: a prognostic model for survival. Ann Oncol. 2014;25(2):467‐471. 10.1093/annonc/mdt537 24355488

[21] Celik E, Semrau R, Baues C, Trommer‐Nestler M, Baus W, Marnitz S. Robot‐assisted extracranial stereotactic radiotherapy of adrenal metastases in oligometastatic non‐small cell lung cancer. Anticancer Res. 2017;37(9):5285‐5291. 10.21873/anticanres.11954 28870966

[22] Oh D, Ahn YC, Seo JM, et al. Potentially curative stereotactic body radiation therapy (SBRT) for single or oligometastasis to the lung. Acta Oncol. 2012;51(5):596‐602. 10.3109/0284186x.2012.681698 22548366

[23] Pastorino U, Buyse M, Friedel G, et al. Long‐term results of lung metastasectomy: prognostic analyses based on 5206 cases. J Thorac Cardiovasc Surg. 1997;113(1):37‐49. 10.1016/s0022-5223(97)70397-0 9011700

[24] Poon I, Erler D, Dagan R, et al. Evaluation of definitive stereotactic body radiotherapy and outcomes in adults with extracranial oligometastasis. JAMA Netw Open. 2020;3(11):e2026312. 10.1001/jamanetworkopen.2020.26312 33196810 PMC7670310

[25] Redmond KJ, Lo SS, Dagan R, et al. A multinational report of technical factors on stereotactic body radiotherapy for oligometastases. Future Oncol. 2017;13(12):1081‐1089. 10.2217/fon-2016-0479 28152619

[26] Dagan R, Lo SS, Redmond KJ, et al. A multi‐national report on stereotactic body radiotherapy for oligometastases: patient selection and follow‐up. Acta Oncol. 2016;55(5):633‐637. 10.3109/0284186X.2015.1118659 27046290

[27] Fine JP, Gray RJ. A proportional hazards model for the subdistribution of a competing risk. Journal of the American Statistical Association. 1999;94(446):496‐509. 10.2307/2670170

[28] R core Team . R: A Language and Environment for Statistical Computing. Vienna, Austria: R Foundation for Statistical Computing. 2020.

[29] Nicosia L, Cuccia F, Mazzola R, et al. Stereotactic body radiotherapy (SBRT) can delay polymetastatic conversion in patients affected by liver oligometastases. J Cancer Res Clin Oncol. 2020;146(9):2351‐2358. 10.1007/s00432-020-03223-9 32356176 PMC11804598

[30] Casiraghi M, De Pas T, Maisonneuve P, et al. A 10‐year single‐center experience on 708 lung metastasectomies: the evidence of the ‘international registry of lung metastases’. J Thorac Oncol. 2011;6(8):1373‐1378. 10.1097/JTO.0b013e3182208e58 21642869

[31] Lievens Y, Guckenberger M, Gomez D, et al. Defining oligometastatic disease from a radiation oncology perspective: an ESTRO‐ASTRO consensus document. Radiother Oncol. 2020;148:157‐166. 10.1016/j.radonc.2020.04.003 32388150

[32] Pasalic D, Lu YI, Betancourt‐Cuellar SL, et al. Stereotactic ablative radiation therapy for pulmonary metastases: improving overall survival and identifying subgroups at high risk of local failure. Radiother Oncol. 2020;145:178‐185. 10.1016/j.radonc.2020.01.010 32044530

[33] Macchia G, Lazzari R, Colombo N, et al. A large, multicenter, retrospective study on efficacy and safety of stereotactic body radiotherapy (SBRT) in oligometastatic ovarian cancer (MITO RT1 study): a collaboration of MITO, AIRO GYN, and MaNGO Groups. Oncologist. 2020;25(2):e311‐e320. 10.1634/theoncologist.2019-0309 32043791 PMC7011643

[34] Dell’Acqua V, Surgo A, Kraja F, et al. Stereotactic radiation therapy in oligometastatic colorectal cancer: outcome of 102 patients and 150 lesions. Clin Exp Metastasis. 2019;36(4):331‐342. 10.1007/s10585-019-09976-z 31165360

[35] Kinj R, Bondiau P‐Y, François E, et al. Radiosensitivity of colon and rectal lung oligometastasis treated with stereotactic ablative radiotherapy. Clin Colorectal Cancer. 2017;16(3):e211‐e220. 10.1016/j.clcc.2016.08.003 27670890

