# Supplementary material for: Late metastatic presentation is associated with improved survival and delayed wide‐spread progression after ablative stereotactic body radiotherapy for oligometastasis
Source: Cancer Med. 2021 Aug 25;10(18):6189–98. doi: 10.1002/cam4.4133 (PMC8446561; doi:10.1002/cam4.4133)
Supplement: Supplementary file 1 — Supplementary Material [file CAM4-10-6189-s001.docx]

**Supplemental Table 1. Multivariable Analysis of Factors Associated with Outcomes in Patients with Prostate Cancer**

|  | OS |  | PFS |  | WSP |  |
| --- | --- | --- | --- | --- | --- | --- |
|  | HR (95% CI) | P value | HR (95% CI) | P value | HR (95% CI) | P value |
| Late Metastasis | 0.38 (0.08-1.73) | 0.21 | 1.26 (0.69-2.31) | 0.45 | 0.75 (0.35-1.62) | 0.47 |
| Total # Metastases | 0.32 (0.07-1.49) | 0.15 | 1.20 (0.77-1.88) | 0.42 | 1.37 (0.78-2.38) | 0.27 |
| Bone Only Metastasis | 0.32 (0.04-2.24) | 0.25 | 0.76 (0.39-1.46) | 0.41 | 0.75 (0.30-1.85) | 0.53 |
| Total PTV Volume | 1.017 (1.004-1.031) | 0.011 | 1.009 (1.004-1.014) | 0.0002 | 1.009 (1.003 -1.010) | 0.0019 |
| Total BED_10_ | 1.004 (0.987-1.022) | 0.62 | 0.994 (0.989-1.000) | 0.039 | 0.995 (0.989-1.000) | 0.12 |
| Gleason Score | 0.77 (0.35-1.68) | 0.51 | 0.79 (0.60-1.05) | 0.1 | 0.74 (0.53-1.03) | 0.073 |
| Pre-SBRT PSA | 0.98 (0.90-1.06) | 0.58 | 0.99 (0.98-1.01) | 0.5 | 0.99 (0.972-1.01) | 0.36 |

Abbreviations: BED_10_, biologically effective dose at α/β ratio of 10Gy; CI, confidence interval; HR, hazard ratio; OS, overall survival; PFS, progression-free survival; WSP, wide-spread progression.

**Supplemental Table 2. Multivariable Analysis of Factors Associated with Outcomes in Patients with Breast Cancer**

|  | OS |  | PFS |  | WSP |  |
| --- | --- | --- | --- | --- | --- | --- |
|  | HR (95% CI) | P value | HR (95% CI) | P value | HR (95% CI) | P value |
| Late Metastasis | 0.44 (0.15-1.27) | 0.13 | 1.12 (0.61-2.06) | 0.71 | 0.74 (0.37-1.48) | 0.39 |
| Total # Metastases | 0.76 (0.40-1.46) | 0.41 | 1.17 (0.82-1.66) | 0.39 | 1.14 (0.75-1.74) | 0.54 |
| Bone Only Metastasis | 0.26 (0.08-0.82) | 0.02 | 1.10 (0.53-2.28) | 0.81 | 0.78 (0.35-1.75) | 0.54 |
| Total PTV Volume | 1.006 (1.002-1.011) | 0.01 | 1.001 (0.998-1.004) | 0.56 | 1.002 (1.000 -1.004) | 0.12 |
| Total BED_10_ | 1.008 (0.997-1.019) | 0.15 | 1.000 (0.992-1.008) | 0.99 | 1.001 (0.994-1.010) | 0.74 |
| Grade 2 | 0.55 (0.09-3.18) | 0.50 | 1.49 (0.40-5.50) | 0.55 | 2.06 (0.35-12.2) | 0.42 |
| Grade 3 | 1.38 (0.25-7.45) | 0.71 | 2.81 (0.78-10.1) | 0.11 | 2.64 (0.43-16.2) | 0.29 |
| Grade Unknown | 3.11 (0.50-19.3) | 0.22 | 3.67 (0.95-14.3) | 0.06 | 4.44 (0.68-28.8) | 0.12 |
| HR- HER2+ | 0.60 (0.16-2.26) | 0.45 | 0.62 (0.27-1.40) | 0.25 | 0.64 (0.26-1.58) | 0.33 |
| Triple Negative | 1.53 (0.39-6.06) | 0.54 | 1.01 (0.36-2.83) | 0.98 | 1.16 (0.47-2.83) | 0.75 |
| Subtype Unknown | 3.39 (0.48-23.7) | 0.22 | 0.63 (0.16-2.54) | 0.51 | 0.27 (0.03-2.18) | 0.22 |

Abbreviations: BED_10_, biologically effective dose at α/β ratio of 10Gy; CI, confidence interval; HR, hazard ratio; OS, overall survival; PFS, progression-free survival; WSP, wide-spread progression.

**Supplemental Figure 1.** Kaplan-Meier curves of overall survival (A), progression-free survival (B) and wide-spread progression (C) among patients with prostate cancer. Dashed lines, late metastatic presentation; solid lines, early metastatic presentation.


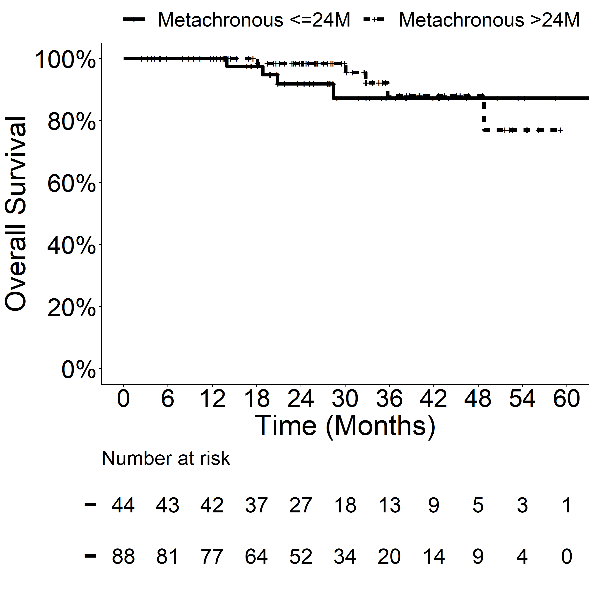

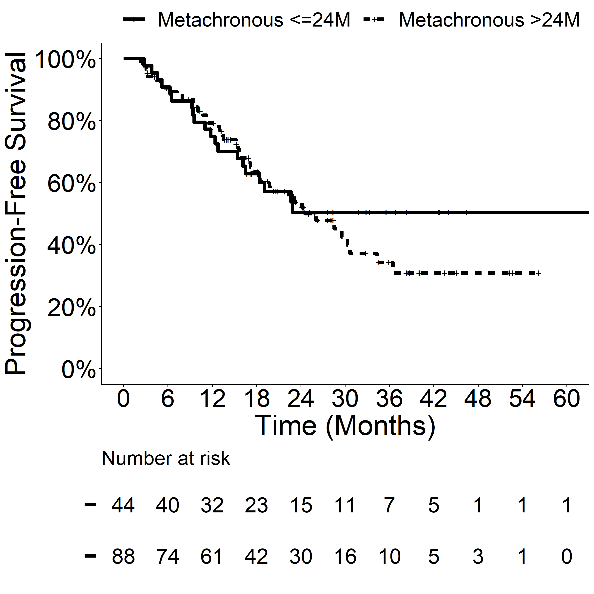

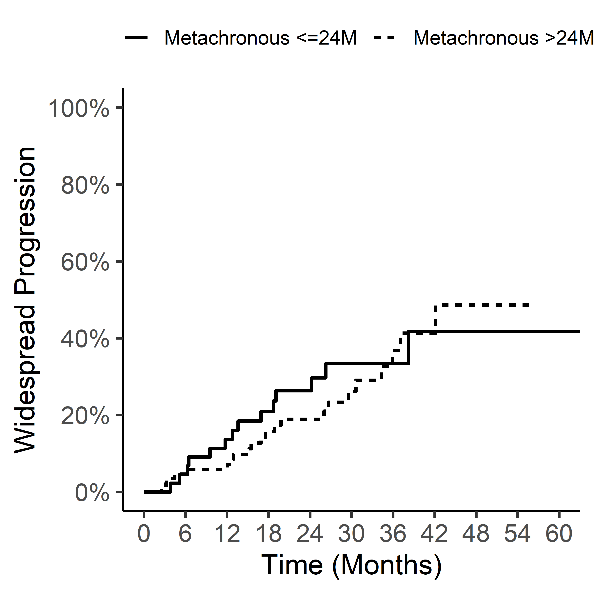


A

B

C

**Supplemental Figure 2.** Kaplan-Meier curves of overall survival (A), progression-free survival (B) and wide-spread progression (C) among patients with breast cancer. Dashed lines, late metastatic presentation; solid lines, early metastatic presentation.


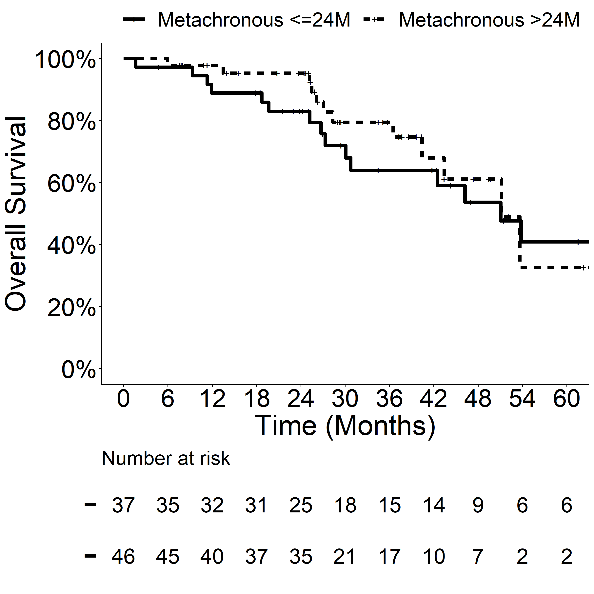

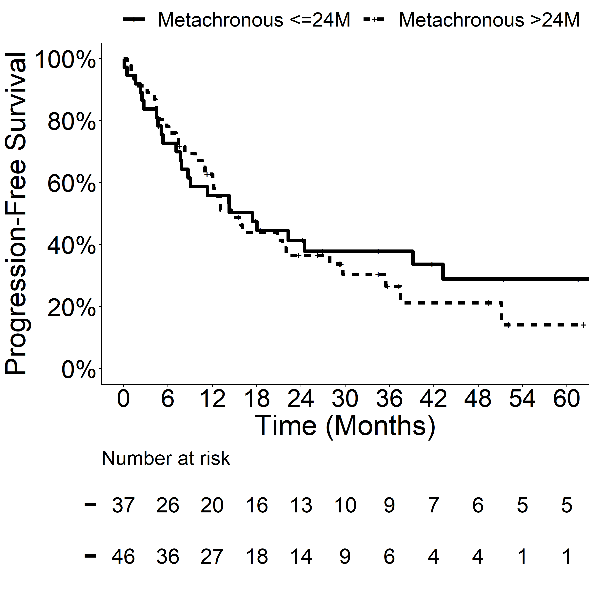

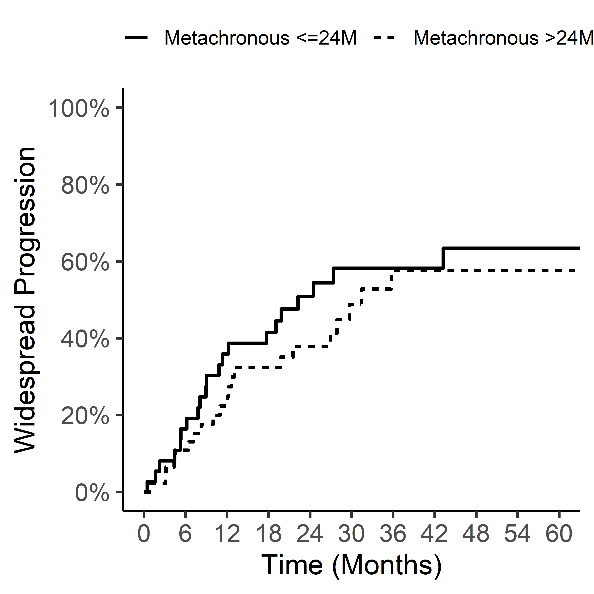


A

B

C

**Supplemental Figure 3.** Kaplan-Meier curves of overall survival in patients with NSCLC (A), colorectal (B), kidney (C), prostate (D) and breast cancer (E), stratified by synchronous (≤6 months of primary diagnosis, red), early metachronous (6-24 months, green) and late metachronous (>24 months, blue) timing. Multivariable p values for early metachronous vs synchronous metastases: NSCLC 0.46, colorectal 0.42, kidney 0.09, prostate 0.86, and breast 0.42.


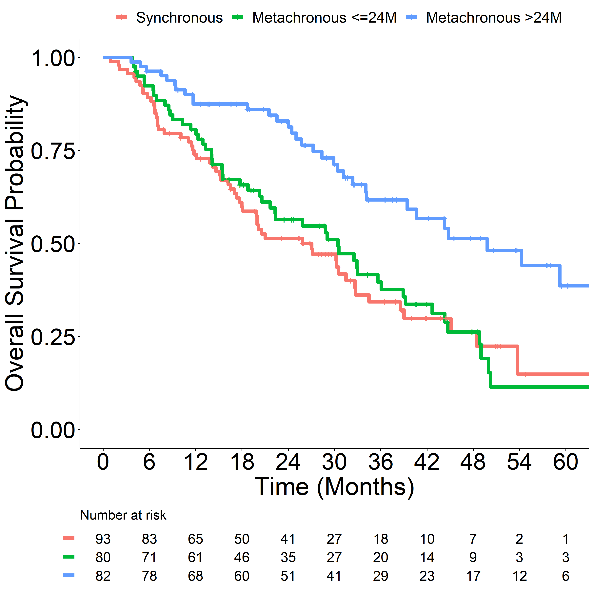

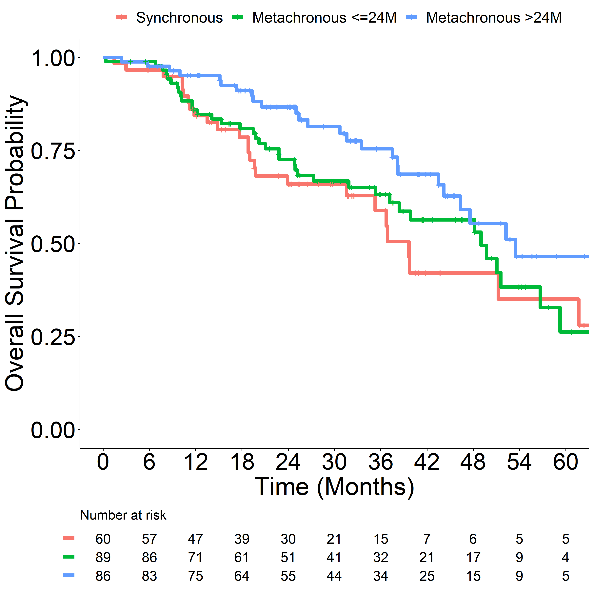

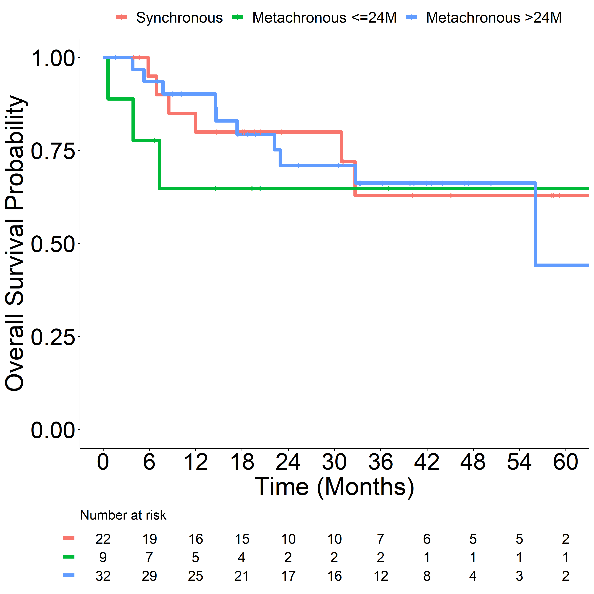

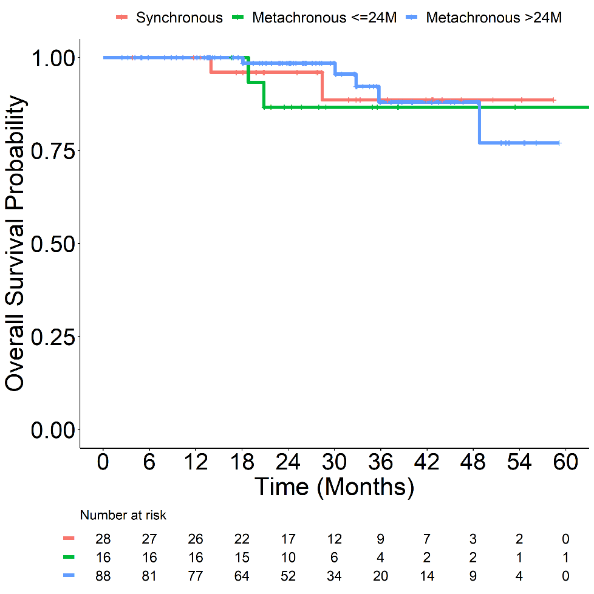

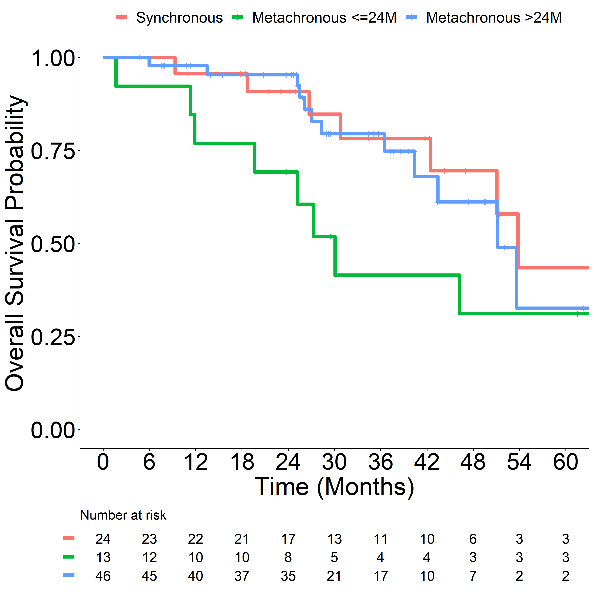


A

B

C

D

E
